# Research on the deep learning-based exposure invariant spectral reconstruction method

**DOI:** 10.3389/fnins.2022.1031546

**Published:** 2022-10-17

**Authors:** Jinxing Liang, Lei Xin, Zhuan Zuo, Jing Zhou, Anping Liu, Hang Luo, Xinrong Hu

**Affiliations:** ^1^School of Computer Science and Artificial Intelligence, Wuhan Textile University, Wuhan, Hubei, China; ^2^Hubei Provincial Engineering Research Center for Intelligent Textile and Fashion, Wuhan, Hubei, China; ^3^Engineering Research Center of Hubei Province for Clothing Information, Wuhan, China

**Keywords:** spectral reconstruction, multispectral image, color science, convolutional neural network, exposure invariant, dense connections

## Abstract

The surface spectral reflectance of an object is the key factor for high-fidelity color reproduction and material analysis, and spectral acquisition is the basis of its applications. Based on the theoretical imaging model of a digital camera, the spectral reflectance of any pixels in the image can be obtained through spectral reconstruction technology. This technology can avoid the application limitations of spectral cameras in open scenarios and obtain high spatial resolution multispectral images. However, the current spectral reconstruction algorithms are sensitive to the exposure variant of the test images. That is, when the exposure of the test image is different from that of the training image, the reconstructed spectral curve of the test object will deviate from the real spectral to varying degrees, which will lead to the spectral data of the target object being accurately reconstructed. This article proposes an optimized method for spectral reconstruction based on data augmentation and attention mechanisms using the current deep learning-based spectral reconstruction framework. The proposed method is exposure invariant and will adapt to the open environment in which the light is easily changed and the illumination is non-uniform. Thus, the robustness and reconstruction accuracy of the spectral reconstruction model in practical applications are improved. The experiments show that the proposed method can accurately reconstruct the shape of the spectral reflectance curve of the test object under different test exposure levels. And the spectral reconstruction error of our method at different exposure levels is significantly lower than that of the existing methods, which verifies the proposed method’s effectiveness and superiority.

## Introduction

The visual system is important for humans to sense the external environment. The eye can sense light radiation in the visible light range in the visual system. The light stimulation photoreceptor cells receive will eventually form a comprehensive vision in the brain. By simulating visual perception, digital cameras use color filter array sensors and image signal processing technology to record the radiation spectrum of natural scenes as a color image that conforms to human visual perception. However, color imaging technology has a limited ability to reproduce colors and characterize objects, and the information contained in the visible spectrum is far beyond the RGB data. The metamerism problem of color imaging is the key issue of its use in high-fidelity color reproduction and high-precision material characterization (Kang, 2006).

It is well known that spectral reflectance is the “fingerprint” of color information, which can effectively overcome the influence of light source and observer on color reproduction and object characterization. It is often used in agriculture, cultural relic protection, skin health monitoring, and other fields (Kim et al., 2017; Xu et al., 2017; Ablet et al., 2019). In addition, in the field of computer vision, multispectral data can improve detection accuracy (Hwang et al., 2015). However, due to the limitations of current multispectral imaging technology, such as the complexity of the systems (Hardeberg et al., 2002; Liang, 2012) and the low spatial resolution (Cucci et al., 2016; Daniel et al., 2016), the current spectral cameras cannot quickly acquire multispectral images with high spatial resolution, which restricts the wide application of multispectral images.

Reconstructing multispectral images of scenes from RGB images has been widely researched in many fields. Spectral reconstruction is one of the ill-conditioned inverse problems (Ribes and Schmitt, 2008). The same RGB data may correspond to completely different spectral reflectance data. In natural scenes, however, there is always a close correlation between the RGB data of an image and the corresponding multispectral image. Based on mathematical modeling, the relationship between RGB data and corresponding multispectral data can be established, and fairly accurate spectral reconstruction results can be obtained (Lin and Finlayson, 2020). Therefore, spectral reconstruction technology is easier to apply to open environments than spectral cameras and quickly acquires high spatial resolution multispectral images.

Current spectral reconstruction methods are mainly divided into two different classes: machine learning and deep learning-based methods (Liang et al., 2016, 2019; Galliani et al., 2017; Liang and Wan, 2017; Shi et al., 2018; Yan et al., 2018; Liang and Xiao, 2020; Zhang et al., 2020). The machine learning-based methods include pseudo-inverse, kernel algorithm, principal component analysis, and so on (Liang et al., 2019). The pseudo-inverse method builds a reconstruction matrix based on the error between the reconstructed and ground-truth spectra of the training data. The kernel algorithm uses the kernel function to transform the response values to the kernel space and then calculates the reconstruction matrix. And the principal component analysis method uses the top k principals and coefficient matrix to reconstruct the spectral reflectance of the target. In summary, the current machine learning-based spectral reconstruction methods are all based on the digital camera imaging model to reconstruct the spectral reconstruction matrix. However, they are all exposure sensitive, and the reconstructed spectral errors are large when applied in non-uniform lighting environments.

**FIGURE 1:**
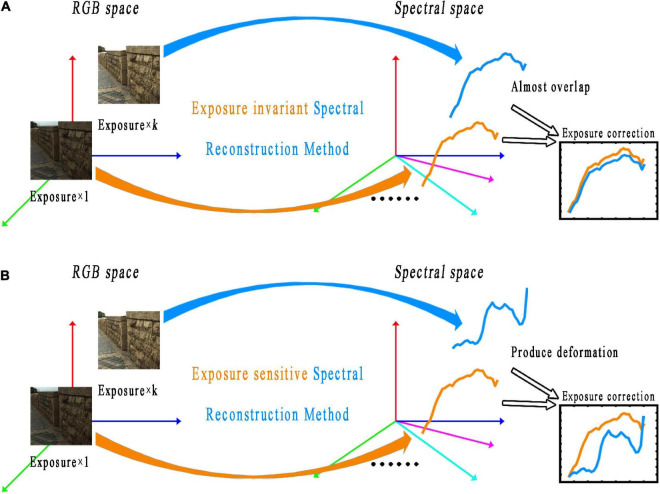
Schematic diagram of the exposure invariant **(A)** and exposure sensitive **(B)** spectral reconstruction methods. The photos appearing in these figures are chosen from the public multispectral dataset from the NTIRE challenges (https://icvl.cs.bgu.ac.il/projects/ntire2020/).

In recent years, with the rapid development of deep learning in the field of computer vision, classical network models, such as convolutional neural networks and generative adversarial networks, have been used in spectral reconstruction. Deep learning-based spectral reconstruction models usually use a large number of data sample pairs as support to establish the mapping relationship between RGB images and multispectral images. For the deep learning-based spectral reconstruction, Yan et al. (2018) applied the U-net network to spectral reconstruction. Galliani et al. (2017) utilized a variant of full convolution for the end-to-end spectral reconstruction task. Shi et al. (2018) proposed a network model based on residuals and densely connected structures (He et al., 2016; Huang et al., 2017). Zhang et al. (2020) proposed a deep learning spectral reconstruction model based on dense connections. However, the existing deep learning-based spectral reconstruction methods with good spectral reconstruction accuracy usually have complex network structures, a large number of parameters, and exposure sensitivity (as shown in Figure 1B). That is, the spectral reconstruction model constructed under one exposure level cannot adapt to another, or the reconstructed spectral curve will deviate from the ground truth.

An optimized deep learning-based spectral reconstruction method is proposed based on a lightweight convolutional neural network (CNN) to address the issues arising from the current deep learning-based spectral reconstruction methods, such as a large number of model parameters and sensitivity to exposure changes of test images. Moreover, some optimized measures are integrated into the network. First, the input RGB training image is randomly multiplied by an exposure adjustment coefficient during model training so that the model can see images with more exposure levels. According to the exposure adjustment coefficient of the training image, the reconstructed multispectral image (RMSI) is reciprocally corrected in the loss function to make the model learn the exposure invariant spectral features that correspond to the training images. Secondly, the dense connection mechanism in the original model not only helps to alleviate the problem of model gradient disappearance but also greatly reduces the number of model parameters. Finally, an attention mechanism is introduced into the model to improve spectral reconstruction accuracy by adaptively weighting the feature channels. Experimental results show that the proposed method not only achieves the performance of exposure invariant but also exhibits better spectral reconstruction accuracy than existing methods.

## Models and methods

### Imaging model

The imaging process of a color digital camera involves three factors, light source, object, and camera. The light sources are usually characterized by their spectral power distribution. When the light source irradiates the surface of the object, the object will selectively absorb some wavelengths of energy and reflect the rest to form a radiance spectral that integrates the light source information and the reflection characteristics of the object. The radiance spectra are focused and incident on the camera sensor through the lens. After photoelectric conversion and analog-to-digital conversion, the radiance spectral forms a raw format digital image on the camera sensor, and then the raw image undergoes a series of image signal processing (dark current correction, dead pixel correction, white balance correction, demosaicing, color space conversion, etc.) to form the visually pleasing color image (Nakamura, 2017).

The imaging mentioned above process of a digital camera can be generally divided into a linear imaging stage and a nonlinear processing stage. The linear imaging stage is from the radiance spectral to the raw image, and the nonlinear imaging stage is from the raw image to the final visual pleased color image. However, because different brands of cameras usually use different image signal processing algorithms, and because they are all the company’s intellectual properties, it is hard to accurately and uniformly express the nonlinear stage. The current research on spectral reconstruction is carried out in the linear imaging mode (Ribes and Schmitt, 2008), as shown in Eq. 1:


(1) $$\begin{array}{c} \begin{array}{c} d_{i}=\int l\,\left(\lambda \right)\,r\,\left(\lambda \right)\,t\,\left(\lambda \right)\,f_{i}\,\left(\lambda \right)\,s\,\left(\lambda \right)\,d\lambda +n_{i}\\ =\int m_{i}\,\left(\lambda \right)\,r\,\left(\lambda \right)\,d\lambda +n_{i}, \end{array} \end{array}$$

where *d*_*i*_ represents the response of the *i*-th channel of a pixel in the image, *l*(λ) is the spectral distribution of the lighting source, *r*(λ) is the spectral reflectance of a point on the surface of the object, *t*(λ) is the overall transmittance of the camera lens optics, *f*_*i*_(λ) is the transmittance of the *i*-th channel filter of the camera, *s*(λ) is the spectral sensitivity function of the camera sensor, λ indicates the wavelength, *n*_*i*_ represents the noise signal of the *i*-th channel of the digital camera, *m_*i*_* = *l*(λ)*t*(λ)*f*_*i*_(λ)*s*(λ) represents the overall spectral sensitivity function of the *i*-th channel of a digital camera (Liang et al., 2019). Eq. 1 can be abbreviated into the matrix form as shown in Eq. 2:


(2) $$\begin{array}{c} d=Mr \end{array}$$

where *d* represents the *K* × 1 dimensional response value vector of a pixel, *K* is the channel number of the imaging system. M is the *K* × *N* dimensional overall sensitivity function matrix of the imaging system that contains *l*(λ), *t*(λ), *f*_*i*_(λ), and *s*(λ), and *r* represents the *N* × 1 dimensional spectral vector of a pixel.

### Spectral reconstruction model

Because of the high correlation between RGB images and the corresponding multispectral images, learning-based methods can be used to model the mapping between RGB and multispectral images. In recent years, with the success of deep learning in many computer vision tasks, CNN based methods have gradually been applied to spectral reconstruction. Assuming that an RGB image and its corresponding multispectral image are given, the mapping between RGB and multispectral image can be described as follows:


(3) $$\begin{array}{c} r=f\,\left(d\right) \end{array}$$

where *d* is the digital response value of any pixel in the RGB image, *r* is the spectral reflectance corresponding to the pixel, and *f*(⋅) is the mapping model from RGB values to multispectral reflectance. When the spectral reconstruction model *f*(⋅) is established, the corresponding spectral reflectance *r* can be obtained by Eq. 3 for any given pixel response value *d*. The multispectral image corresponding to the RGB image is obtained.

### Proposed optimized method

As mentioned in the introduction section, neural networks can learn the mapping relationship from RGB images to multispectral images, and convolutional networks and generative adversarial networks are gradually being used in spectral reconstruction. However, existing deep learning-based spectral reconstruction models usually need to train millions of model parameters. And during model training, convolution operators are used to extract deep image features. Multiple sets of convolution operators are usually superimposed to improve the network’s performance, making the deep learning spectral reconstruction methods more complex than machine learning-based ones.

In addition, although the accuracy of the existing machine learning-based spectral reconstruction method is limited by the appropriate design of the spectral reconstruction model [such as the use of root polynomial extended regression (Lin and Finlayson, 2019)] and using linear raw image data, the method can achieve the ability of exposure invariant (as shown in Figure 1A). Unlike machine learning-based methods, which can easily perform linear regression, deep learning-based spectral reconstruction methods are all nonlinear mathematical models. This is because, to ensure the learning ability and generalization performance of the deep learning model, the nonlinear activation functions (such as *Relu*, *Prelu*, and *Sigmoid* functions) and non-zero bias terms are always included in the model. Therefore, obtaining the corresponding linear output is difficult for the existing deep learning-based spectral reconstruction model when a set of linear inputs is given. In other words, the existing deep learning-based spectral reconstruction models are exposure sensitive and cannot guarantee the correctness of the reconstructed spectral curve (as shown in Figure 1B). Take the typical architecture of a single neuron in the deep learning framework as an example, as shown in Eq. 4:


(4) $$\begin{array}{c} y=h\,\left(w^{T}\,x+b\right) \end{array}$$

where *x* is the input, *w* is the weight, *b* is the bias, *y* is the output, and *h*(⋅) is the activation function. Without considering the influence of exposure change on spectral reconstruction, the corresponding output y can always be obtained for any given input x. However, considering the exposure influence on spectral reconstruction (Lin and Finlayson, 2019), the output *ky* is difficult to acquire directly for the input *kx* for current deep learning-based spectral reconstruction methods, where *k* is the exposure adjustment coefficient.

Therefore, for the existing deep learning-based spectral reconstruction model, how to make it exposure invariant, as shown in Eq. 5, will be the key problem when using it in an open environment with variable and non-uniform illumination (Liang and Xiao, 2020). In addition, it is also important to reconstruct high-precision multispectral images in an open illumination environment:


(5) $$\begin{array}{c} k\,r=f\,\left(k\,d\right) \end{array}$$

Based on the statements above, to build a lightweight deep learning-based spectral reconstruction model with the ability to be exposure invariant and, at the same time, improve the spectral reconstruction accuracy, in this study, an optimized deep learning-based spectral reconstruction method is proposed by referencing the existing models. The ability to be exposure invariant is first achieved for the spectral reconstruction model through training data enhancement. Then, the attention mechanism of spectral reconstruction is further introduced into the model to improve the spectral reconstruction accuracy. Details of the proposed optimized method are described as follows.

**FIGURE 2:**
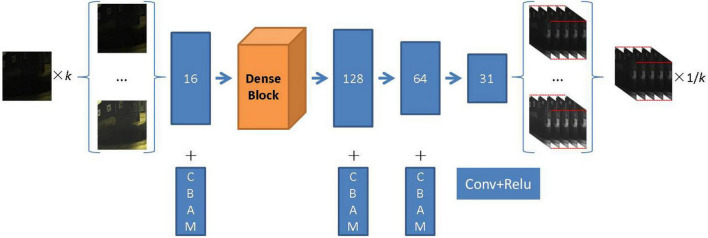
Schematic diagram of training the deep learning-based spectral reconstruction model based on data augmentation. The photos appearing in these figures are chosen from the public multispectral dataset from the NTIRE challenges (https://icvl.cs.bgu.ac.il/projects/ntire2020/).

The proposed method is based on the neural network model developed by Zhang et al. (2020). In the training stage, the input RGB image is first randomly multiplied by the exposure adjustment coefficient *k* to simulate the exposure change. Secondly, to keep the RMSI exposure invariant, the RMSI is multiplied by the reciprocal of the exposure adjustment coefficient in the loss function. Finally, various evaluation metrics are calculated using the RMSI and the ground truth multispectral image (GMSI). The training process is shown in Figure 2.

In addition, the attention mechanism is introduced into the original model to further improve the spectral reconstruction accuracy. Currently, the commonly used attention modules include channel attention, spatial attention, and non-local networks (Hu et al., 2018; Wang et al., 2018; Woo et al., 2018). If the feature maps of each layer in the deep learning network are fused into a total feature map, the weights of the feature maps of each dimension relative to the total feature map are all different. The attention mechanism is to learn the weight of each feature map relative to the total feature map and then generate a weight mask and weight the original feature map to realize the effective use of feature information.

For the existing attention modules, Hu et al. (2018) proposed a compressed excitation network, which has won the championship in the ImageNet competition image classification track, and its structure mainly includes compression (Squeeze), excitation (Excitation), and feature weighting (Scale) modules. The author first uses the pooling operation to compress the feature map space and then outputs a real number on each channel to extract the channel dimension information. Secondly, the activation function will generate weights for each feature channel through the excitation module, including the fully connected layer and the activation function. Finally, the initial feature map is scaled using the weight mask, and the re-calibration of the original feature is completed on the feature channel. The spatial attention is to compress the channel information of the feature map and excite it in the spatial dimension. The mask of the spatial domain is calculated by compressing the channel, and the mask is multiplied by the original feature value. The non-local network can directly calculate the relationship between any two positions on the feature image, but the network will generate more parameters in the spectral reconstruction task.

**FIGURE 3:**
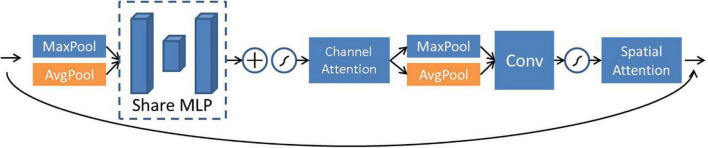
Schematic diagram of convolutional block attention module (CBAM) attention module.

The convolutional block attention module (CBAM) is a tandem hybrid attention module (Woo et al., 2018). It learns the attention of the two dimensions in turn according to the order of the channel domain attention and the spatial domain attention. CBAM can be used as a plug-and-play module in neural networks and is one of the most commonly used attention mechanism algorithms in the field of computer vision research. The major difference between the CBAM module and the SE (Hu et al., 2018) module is that a parallel max pooling layer is added, and the attention mechanism is learned for more than two domains, such as the spatial domain and the channel domain, which makes its feature extraction more sufficient. This article adopts the CBAM module and adds a residual structure to it. The CBAM attention module is shown in Figure 3.

The feature channel information is first compressed into a real number through the average pooling and max pooling layers for the channel attention module. Then the feature weights are extracted through two fully connected layers, including the activation function. Among the fully connected layers, the first fully connected layer can reduce the feature dimension to 1/s of the input, where *s* is the compression parameter. In this article, the values of s are 4, 32, and 16, respectively. After the feature map output is by the first fully connected layer, it is activated by the Relu function and restored to the original dimension by the second fully connected layer. The Sigmoid function processes the feature information processed by the second fully connected layer. At this stage, the weight calibration of the original features is completed. Then, the spatial attention mechanism module takes the output of channel attention as input and uses average pooling and maximum pooling to integrate channel feature information. After that, the two parts of the features are combined using 1 × 1 convolution for dimensionality reduction, and finally, the required mask is acquired through the Sigmoid activation function. The overall expression for CBAM attention is expressed as Eqs 6, 7:


(6) $$\begin{array}{c} C=\delta \,\left(M\,L\,P\,\left(A\,\text{vg}\,P\,o\,o\,l\,\left(x\right)+M\,a\,x\,P\,o\,o\,l\,\left(x\right)\right)\right) \end{array}$$

(7) $$\begin{array}{c} S=\delta \,\left(f\,\left(A\,\text{vg}\,P\,o\,o\,l\,\left(x\right);M\,a\,x\,P\,o\,o\,l\,\left(x\right)\right)\right), \end{array}$$

where *x*, δ, and *MLP* are the input, activation function, and multilayer perceptron, respectively. *C* is the output channel attention feature, and *S* is the output spatial attention feature. Based on Eqs 6, 7, the weight of each channel can be adaptively learned and used to weigh the corresponding channel features to improve spectral reconstruction accuracy.

This is plotted in Figure 2 of the overall architecture of the proposed deep learning-based spectral reconstruction model. For an RGB image of any input, the feature information of the shallow layer of the input image is first extracted through 16 layers of convolution and then through 7 layers of dense connection. Each dense connection layer has 16 convolution kernels. The dense structure further improves the reuse rate of channel features compared with the residual structure. The size of the feature map remains unchanged, and the number of channels gradually increases with the deepening of the network, which greatly reduces the parameters of the network. Dense connections also mitigate the vanishing gradient problem to some extent.

After shallow feature extraction and a densely connected network, a feature information map with 128 layers is obtained, which is input into the reconstruction layer that includes three layers of convolution. The kernel size of each layer of convolution in the network is set at 3, the activation function is Relu, and the CBAM module is added to the shallow feature extraction and reconstruction layer to further improve the robustness of the network. Finally, we obtain the RMSI corresponding to the input of the RGB image.

## Experiment

### Experiment settings

To test the effectiveness and superiority of the proposed method, we carried out the verification experiment. The proposed deep learning-based spectral reconstruction framework is implemented in TensorFlow and is trained using the platform of Intel Xeon and Tesla V100. The database for the experiments is NTIRE2018 (Arad et al., 2018), which is extended from the ICVL dataset (Arad and Ben-Shahar, 2016). The ICVL dataset consists of 203 multispectral images captured with the hyperspectral camera Specim PS Kappa DX4. The spatial resolution of each image is 1392 × 1300 pixels, and the spectral sampling range of each multispectral image is from 400 to 700 nm with a sampling interval of 10 nm.

In addition, in the NTIRE2018 challenge, 53 multispectral images with the same spatial and spectral resolution were added to further expand the dataset, so the experiment finally used 256 multispectral images as training data in this per, and their corresponding RGB images were acquired using the same method as NTIRE2018 and NTIRE2020 (Arad et al., 2018, 2020). During model training, the data blocks with a size of 40 × 40 pixels are cropped from the training data as input. The learning rate was initially set to 0.0001 and exponentially decayed to a rate of 0.99. The max epoch number was set to 50, and other hyperparameters in the model Zhang et al. (2020) remained unchanged.

### Evaluation metrics

In the experiment, the spectral root-mean-square error (RMSE), the mean relative absolute error (MRAE), and the spectral angle mapping (SAM) error are used to evaluate and compare the spectral reconstruction accuracy of different models. The smaller the value of the evaluation metrics, the closer the RMSI is to the ground truth and the better the performance of the method. Calculation of the evaluation metrics is shown in Equs 8–10, where *n* represents the spectral bands, *i* represents a pixel in a multispectral image, *I*_*R*_ represents the reconstructed multispectral images, and *I*_*G*_ represents the ground-truth multispectral image.


(8) $$\begin{array}{c} M\,R\,A\,E=\frac{1}{n}\,\sum_{i=1}^{n}\left(\left|I_{R}^{\left(i\right)}-I_{G}^{\left(i\right)}\right|/I_{G}^{\left(i\right)}\right) \end{array}$$

(9) $$\begin{array}{c} R\,M\,S\,E=\sqrt{\frac{1}{\text{n}}\,\sum_{i=1}^{n}{\left(I_{R}^{\left(i\right)}-I_{G}^{\left(i\right)}\right)}^{2}} \end{array}$$

(10) $$\begin{array}{c} S\,A\,M=\frac{1}{n}\,{\text{cos}}^{-1}\,\left(\sum_{i=1}^{n}\frac{{\left(I_{R}^{\left(i\right)}\right)}^{T}\,I_{G}^{\left(i\right)}}{{∥I_{R}^{\left(i\right)}∥}_{2}\,{∥I_{G}^{\left(i\right)}∥}_{2}}\right) \end{array}$$

## Results and discussion

### Comparison of spectral reconstruction methods

Using the experimental conditions mentioned above, we tested the effect of the proposed deep learning-based spectral reconstruction method compared with several current advanced methods, such as Yan et al. (2018), Galliani et al. (2017), Zhang et al. (2020), and HSCNN+(Shi et al., 2018). The experimental results are summarized in Tables 1–3, respectively. Where the expression of Exposure × *k* means the exposure adjustment coefficient k adjusts the exposure level of the test image before it is fed into the framework. The value of *k* is used in 1, 0.25, 0.5, 2, and 4 during the testing stage. Additionally, when calculating the spectral reconstruction error, the RMSI is corrected to 1/*k* of the output.

**TABLE 1:** Comparison of the mean relative absolute error (MRAE) (%) of different methods under different tested exposure levels.

	Exposure ×1	Exposure ×0.25	Exposure ×0.5	Exposure ×2	Exposure ×4	*Ave.*	*Std.*
Yan	0.38	8.42	3.97	0.72	1.18	2.93	3.38
Galliani	0.38	6.55	2.02	0.81	1.09	2.17	2.52
Zhang	0.31	6.06	2.01	0.69	0.92	2.00	2.36
HSCNN+	**0.24**	5.70	1.94	0.67	1.15	1.94	2.19
Ours	0.36	**0.42**	**0.37**	**0.36**	**0.38**	**0.38**	**0.03**

The bold values indicate the best results with the smallest spectral reconstruction errors.

**TABLE 2:** Comparison of the mean relative absolute error (MRAE) (%) of different methods under different tested exposure levels.

	Exposure ×1	Exposure ×0.25	Exposure ×0.5	Exposure ×2	Exposure ×4	*Ave.*	*Std.*
Yan	2.43	70.20	26.37	5.27	8.02	22.46	28.28
Galliani	2.49	58.74	17.84	5.79	7.92	18.56	23.18
Zhang	1.85	47.56	16.61	5.09	6.94	15.61	18.69
HSCNN+	**1.39**	51.81	15.50	4.86	8.00	16.31	20.52
Ours	2.28	**2.64**	**2.31**	**2.32**	**2.61**	**2.43**	**0.18**

The bold values indicate the best results with the smallest spectral reconstruction errors.

**TABLE 3:** Comparison of the spectral angle mapping (SAM) errors of different methods under different tested exposure levels.

	Exposure ×1	Exposure ×0.25	Exposure ×0.5	Exposure ×2	Exposure ×4	*Ave.*	*Std.*
Yan	1.47	24.98	15.71	3.66	5.68	10.30	9.85
Galliani	1.55	38.86	11.63	3.98	5.28	12.26	15.33
Zhang	1.29	26.52	10.98	3.63	4.99	9.48	10.17
HSCNN+	**0.99**	15.88	8.62	3.56	5.81	6.97	5.72
Ours	1.56	**1.83**	**1.60**	**1.57**	**1.76**	**1.66**	**0.12**

The bold values indicate the best results with the smallest spectral reconstruction errors.

It can be seen from the experimental results summarized in Tables 1–3 that although the method of HSCNN + showed the smallest reconstruction error when the exposure adjustment coefficient was equal to 1, where the corresponding errors of RMSE, MRAE, and SAM are 0.24, 1.39, and 0.99% when the exposure adjustment coefficient *k* is equal to 0.25, 0.5, 2, and 4. The proposed method is significantly better than the HSCNN+ and other methods. The overall average spectral reconstruction error of all the tested exposure levels is 0.38, 2.43, and 1.66%, respectively, which is significantly better than the existing advanced deep learning spectral reconstruction algorithms.

In addition, according to the standard deviation of each method under different test exposure levels, there is no significant difference in the spectral reconstruction error of the proposed method under different test exposure levels. However, the standard deviation of the compared methods under different tested exposure levels is quite large. On the one hand, the experimental results in Tables 1–3 show that the existing deep learning-based spectral reconstruction model cannot expose invariant. On the other hand, it also proves that the proposed method not only achieves the ability of exposure invariance but also its superiority to the existing methods in spectral reconstruction accuracy. Furthermore, the difference between the proposed and compared methods is insignificant when the test exposure level is equal to 1, which generally proves the effectiveness and superiority of this article’s proposed deep learning-based spectral reconstruction model. Moreover, it should be noted that among the several compared spectral reconstruction algorithms in this article, the accuracy of the HSCNN+ model is generally better than all the other methods.

**FIGURE 4:**
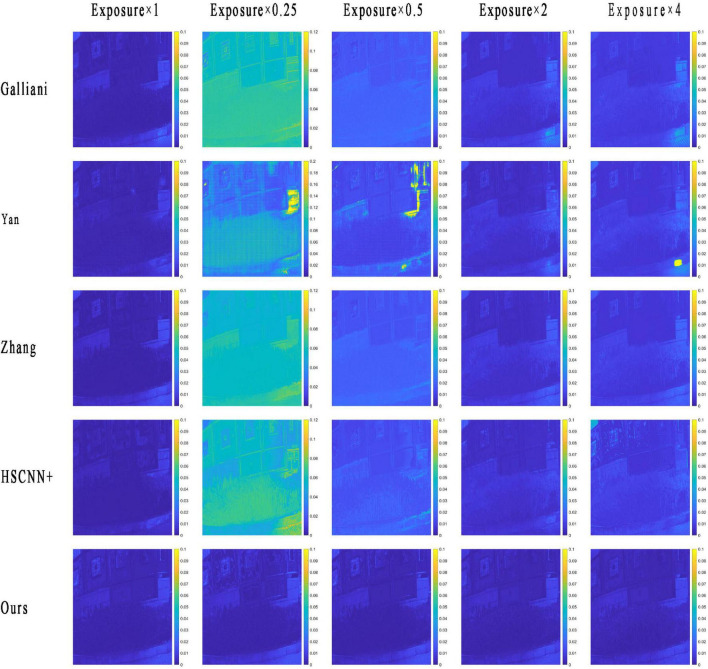
The root-mean-square error (RMSE) map of a reconstructed multispectral image (RMSI) of different methods under different tested exposure levels. The photos appearing in these figures are chosen from the public multispectral dataset from the NTIRE challenges (https://icvl.cs.bgu.ac.il/projects/ntire2020/).

The RMSE map of the RMSI and the GMSI of each method are plotted in Figure 4. The yellower the image color, the greater the spectral reconstruction error, as indicated by the color bar, and the bluer the color, the smaller the error.

**FIGURE 5:**
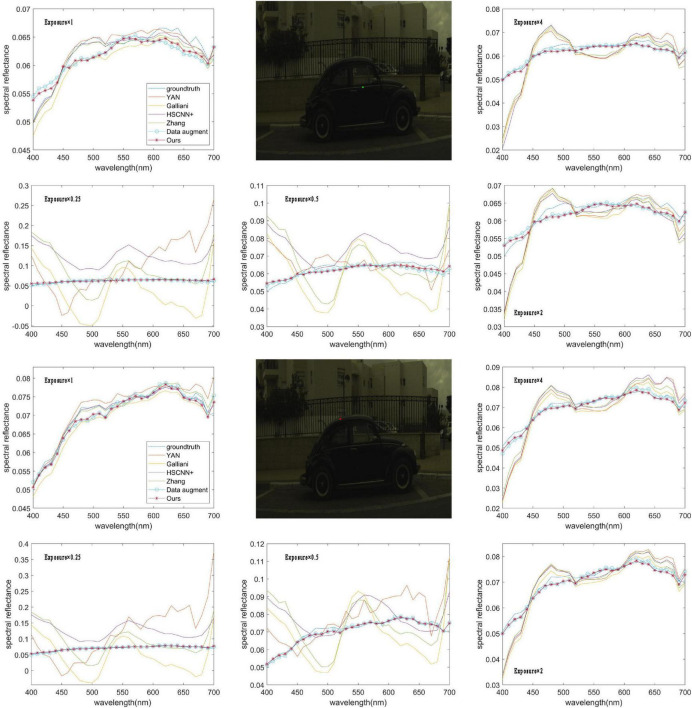
Two reconstructed spectral reflectances under different exposure levels using a different method. The photos appearing in these figures are chosen from the public multispectral dataset from the NTIRE challenges (https://icvl.cs.bgu.ac.il/projects/ntire2020/).

From the results in Figure 4, we can see that the RMSE map of the RMSI and the ground truth by each method are consistent with the objective data in Table 1. When the exposure level of the tested image is equal to 1, the HSCNN+ method performs the best, and the error values in the RMSE map are lower than those of the proposed method. However, in terms of the other four tested exposure levels, the proposed method shows the best spectral reconstruction accuracy, and the four compared methods show very significant spectral reconstruction errors. To further compare the performance of the methods, two reconstructed spectral reflectances under different testing exposure levels using the different methods are plotted in Figure 5. It is easy to find that the spectral reflectance curves reconstructed using the proposed method are closer to the ground truth under different testing exposure levels, while the compared methods only performed well when the exposure level was 1 and performed badly under other testing exposure levels.

**FIGURE 6:**
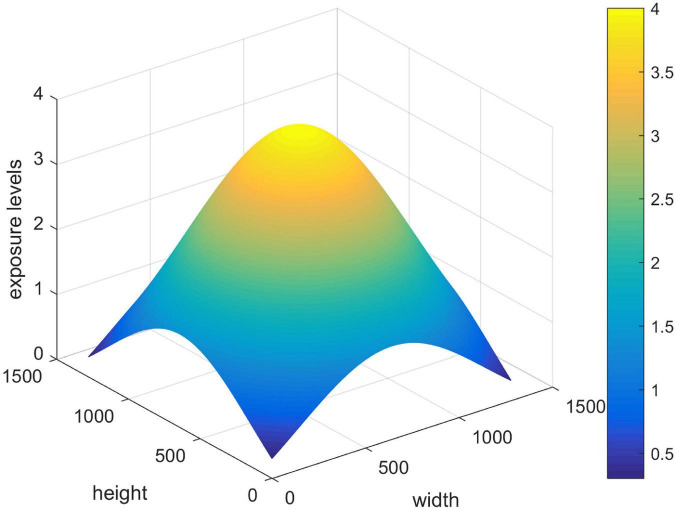
The illumination distribution of non-uniformity points to the light source.

In addition, the verification of the proposed method in reconstructing the non-uniformity illuminated images is also tested. As shown in Figure 6, the non-uniformity point light source is simulated to illuminate an image. Table 4 shows that the image illuminated by a non-uniformity point light source is also well reconstructed. The results in Figures 5, 6 once again prove the effectiveness and superiority of the proposed method.

**TABLE 4:** Comparison of the spectral reconstruction errors of different methods under the illumination of non-uniformity point light source.

	Galliani	YAN	HSCNN+	Zhang	Ours
RMSE	0.85	0.76	0.73	0.72	**0.36**
MRAE	6.21	5.63	5.41	5.42	**2.34**
SAM	4.21	3.89	3.90	3.86	**1.59**

The bold values indicate the best results with the smallest spectral reconstruction errors.

### Analysis of the proposed method

The spectral reconstruction errors of the original method developed by Zhang et al. (2020), the optimized method with only the data enhancement, and the proposed method with data enhancement and attention mechanism, are summarized in Tables 5–7. It is easy to infer from Tables 5–7 that when the tested exposure level is equal to 1, the original method shows the best spectral reconstruction results; however, when the tested exposure level changes to other values, the spectral reconstruction error increases significantly when the data augmentation and attention mechanisms are introduced into the proposed method. Although the spectral reconstruction error increases slightly when the tested exposure level is equal to 1, the new method generally has achieved the ability of exposure invariant, as shown in Figure 1A and Eq. 5.

**TABLE 5:** Comparison of root-mean-square error (RMSE) (%) of different optimization methods under different tested exposure levels.

	Exposure ×1	Exposure ×0.25	Exposure ×0.5	Exposure ×2	Exposure ×4	*Ave.*	*Std.*
Zhang	**0.31**	6.06	2.01	0.69	0.92	2.00	2.36
Data augment	0.36	0.43	0.39	0.37	0.39	0.39	0.27
Ours	0.36	**0.42**	**0.37**	**0.36**	**0.38**	**0.38**	**0.25**

The bold values indicate the best results with the smallest spectral reconstruction errors.

**TABLE 6:** Comparison of mean relative absolute error (MRAE) (%) of different optimization methods under different tested exposure levels.

	Exposure ×1	Exposure ×0.25	Exposure ×0.5	Exposure ×2	Exposure ×4	*Ave.*	*Std.*
Zhang	**1.85**	47.56	16.61	5.09	6.94	15.61	18.69
Data augment	2.30	2.72	2.53	2.49	2.73	2.55	0.18
Ours	2.28	**2.64**	**2.31**	**2.32**	**2.61**	**2.43**	**0.18**

The bold values indicate the best results with the smallest spectral reconstruction errors.

**TABLE 7:** Comparison of spectral angle mapping (SAM) errors of different optimization methods under different tested exposure levels.

	Exposure ×1	Exposure ×0.25	Exposure ×0.5	Exposure ×2	Exposure ×4	*Ave.*	*Std.*
Zhang	**1.29**	26.52	10.98	3.63	4.99	9.48	10.17
Data augment	1.57	1.84	1.72	1.71	1.86	1.74	0.12
Ours	1.56	**1.83**	**1.60**	**1.57**	**1.76**	**1.66**	**0.12**

The bold values indicate the best results with the smallest spectral reconstruction errors.

In addition, although the improvement in the spectral reconstruction accuracy is not so significant when introducing the attention mechanism into the proposed network, the spectral reconstruction accuracy of the proposed method does improve at some specific tested exposure levels. For example, at the tested exposure level of 0.5, the error of the evaluation metric MRAE is reduced by about 8% compared with the only use of data enhancement in the proposed method. At the tested exposure level of 2, the error of the evaluation metric MRAE is reduced by about 6% compared with the pure data enhancement. Moreover, at the tested exposure level of 4, the error of the evaluation metric SAM is reduced by about 5% compared with the pure data enhancement in the proposed method. The proposed method may be further improved based on data enhancement.

**FIGURE 7:**
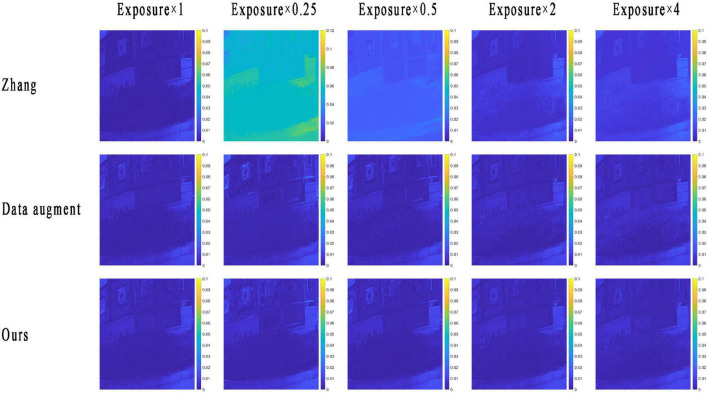
The root-mean-square error (RMSE) map of the reconstructed multispectral image (RMSI) of the original and proposed under different tested exposure levels. The photos appearing in these figures are chosen from the public multispectral dataset from the NTIRE challenges (https://icvl.cs.bgu.ac.il/projects/ntire2020/).

Figure 7 shows the RMSE map of different methods of an original method developed by Zhang et al. (2020), the optimized method with only the data enhancement, and the proposed method with both data enhancement and an attention mechanism. It can be seen from the results in Figure 7 that the introduction of a data enhancement mechanism into the original model can make the method achieve the ability of exposure invariant for spectral reconstruction. And when the attention mechanism is introduced into the proposed method, the spectral reconstruction accuracy can be further improved, but the overall improvement is not obvious, and further optimization measures to improve the spectral reconstruction accuracy can be considered in future studies.

**FIGURE 8:**
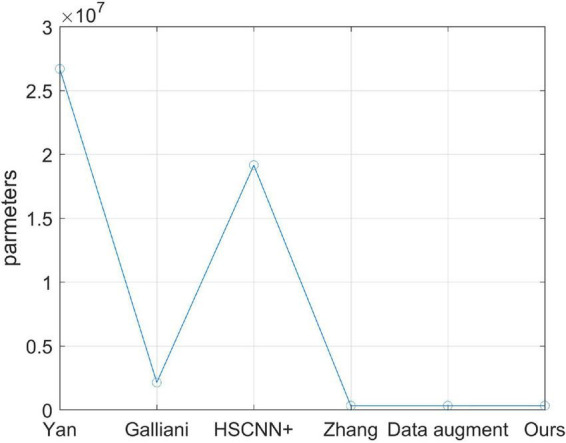
Comparison of the number of learning parameters of different models.

At last, the number of learning parameters for each model in this article is counted and plotted in Figure 8. It is easy to infer from Figure 8 that the number of learning parameters of the original model proposed by Zhang et al. (2020), as well as the optimized model in this article, is very small compared with other models, which means that the proposed method is easy to distribute for practice using.

## Conclusion

Multispectral image acquisition is the prerequisite for its applications. In view of the problems of the existing deep learning-based spectral reconstruction methods, such as a large number of parameters and the exposure invariant. An optimized lightweight neural network for spectral reconstruction is proposed in this article, and the data augmentation and attention mechanisms are introduced into the original method to make it more efficient and exposure invariant. The optimization of the proposed method makes it more robust in practical applications in an open environment with variable light sources and non-uniformity illumination. The shape of the reconstructed spectral reflectance curve of the target can be well preserved using the proposed method under different exposure levels, which provides the foundation for high-precision multispectral image acquisition in an open environment. However, improving the module attention mechanism proposed in the proposed method does not significantly improve the spectral reconstruction accuracy. More research will be carried out to further improve the method in the future.

## Data availability statement

The original contributions presented in this study are included in the article/supplementary material, further inquiries can be directed to the corresponding author.

## Author contributions

JL: methodology, data collection and analysis, and writing-reviewing. LX: methodology, data collection and analysis, and writing. ZZ: data collection and analysis. JZ and AL: data collection. HL: data analysis. XH: methodology, funding acquisition, and writing-reviewing. All authors contributed to the article and approved the submitted version.

## References

[B1] Ablet E. Maimaitiaili B. Sawut M. Shenqun A. (2019). Combined estimation of chlorophyll content in cotton canopy based on hyperspectral parameters and back propagation neural network. *Acta Opt. Sin.* 39:0930003. 10.3788/aos201939.0930003

[B2] Arad B. Ben-Shahar O. (2016). “Sparse recovery of hyperspectral signal from natural RGB images,” in *Proceedings of the European Conference on Computer Vision*, (Cham: Springer), 19–34. 10.1007/978-3-319-46478-7_2

[B3] Arad B. Ben-Shahar O. Timofte R. Gool L. V. Yang M. H. (2018). “NTIRE 2018 challenge on spectral reconstruction from RGB Images,” in *Proceedings of the 2018 IEEE/CVF Conference on Computer Vision and Pattern Recognition Workshops (CVPRW)*, (Salt Lake City, UT: IEEE).

[B4] Arad B. Timofte R. Ben-Shahar O. Lin Y. T. Finlayson G. D. (2020). “Ntire 2020 challenge on spectral reconstruction from an the rgb image,” in *Proceedings of the IEEE/CVF conference on computer vision and pattern recognition workshops*, Piscataway, NJ, 446–447.

[B5] Cucci C. Delaney J. K. Picollo M. (2016). Reflectance hyperspectral imaging for investigation of works of art: Old master paintings and illuminated manuscripts. *Acc. Chem. Res.* 49 2070–2079. 10.1021/acs.accounts.6b00048 27677864

[B6] Daniel F. Mounier A. Pérez-Arantegui J. Pardos C. Prieto-Taboada N. de Vallejuelo S. F. O. (2016). Hyperspectral imaging was applied to the analysis of Goya paintings in the Museum of Zaragoza (Spain). *Microchem. J.* 126 113–120.

[B7] Galliani S. Lanaras C. Marmanis D. Baltsavias E. Schindler K. (2017). Learned spectral super-resolution. *arXiv* [Preprint] 10.48550/arXiv.1703.09470 35895330

[B8] Hardeberg J. Y. Schmitt F. J. Brettel H. (2002). Multispectral color image capture using a liquid crystal tunable filter. *Opt. Eng.* 41 2532–2548.

[B9] He K. Zhang X. Ren S. Sun J. (2016). “Deep residual learning for image recognition,” in *Proceedings of the IEEE conference on computer vision and pattern recognition*, Las Vegas, NV, 770–778. 10.1109/cvpr.2016.90

[B10] Hu J. Shen L. Sun G. (2018). “Squeeze-and-excitation networks,” in *Proceedings of the IEEE conference on computer vision and pattern recognition*, Salt Lake City, UT, 7132–7141. 10.1109/cvpr.2018.00745

[B11] Huang G. Liu Z. Van Der Maaten L. Weinberger K. Q. (2017). “Densely connected convolutional networks,” in *Proceedings of the IEEE conference on computer vision and pattern recognition*, Honolulu, HI, 4700–4708.

[B12] Hwang S. Park J. Kim N. Choi Y. So Kweon I. (2015). “Multispectral pedestrian detection: Benchmark dataset and baseline,” in *Proceedings of the IEEE conference on computer vision and pattern recognition*, Boston, MA, 1037–1045. 10.1109/cvpr.2015.7298706

[B13] Kang H. R. (2006). *Computational color technology.* Bellingham: Spie Press, 155–157.

[B14] Kim T. Visbal-Onufrak M. A. Konger R. L. Kim Y. L. (2017). Data-driven imaging of tissue inflammation using RGB-based hyperspectral reconstruction toward personal monitoring of dermatologic health. *Biomed. Opt. Express* 8 5282–5296. 10.1364/boe.8.005282 29188120 PMC5695970

[B15] Liang H. (2012). Advances in multispectral and hyperspectral imaging for archaeology and art conservation. *Appl. Phys. A* 106 309–323.

[B16] Liang J. Wan X. (2017). Optimized method for spectral reflectance reconstruction from camera responses. *Opt. Express* 25 28273–28287. 10.1364/oe.25.028273

[B17] Liang J. Wan X. Liu Q. Li C. Li J. (2016). Research on filter selection method for broadband spectral imaging system based on ancient murals. *Color Res. Appl.* 41 585–595.

[B18] Liang J. Xiao K. (2020). “Digital camera-based spectral estimation in open environment based on imaging condition correction,” in *Proceedings of the color and imaging conference. Society for imaging science and technology*, 347–350. 10.2352/issn.2169-2629.2020.28.55

[B19] Liang J. Xiao K. Pointer M. R. Wan X. Li C. (2019). Spectra estimation from raw camera responses based on adaptive local-weighted linear regression. *Opt. Express* 27 5165–5180. 10.1364/oe.27.005165 30876119

[B20] Lin Y. T. Finlayson G. D. (2019). “Exposure invariance in spectral reconstruction from rgb images,” in *Proceedings of the color and imaging conference. Society for imaging science and technology*, 284–289. 10.2352/issn.2169-2629.2019.27.51

[B21] Lin Y. T. Finlayson G. D. (2020). “Physically plausible spectral reconstruction from RGB images,” in *Proceedings of the IEEE/CVF Conference on Computer Vision and Pattern Recognition Workshops*, Seattle, WA, 532–533. 10.1109/cvprw50498.2020.00274

[B22] Nakamura J. (ed.) (2017). *Image sensors and signal processing for digital still cameras.* Boca Raton FL: CRC Press.

[B23] Ribes A. Schmitt F. (2008). Linear inverse problems in imaging. *IEEE Signal Process. Mag.* 25 84–99.

[B24] Shi Z. Chen C. Xiong Z. Liu D. Wu F. (2018). “HSCNN+: Advanced cnn-based hyperspectral recovery from rgb images,” in *Proceedings of the IEEE Conference on Computer Vision and Pattern Recognition Workshops*, Salt Lake City, UT, 939–947. 10.1109/cvprw.2018.00139

[B25] Wang X. Girshick R. Gupta A. He K. (2018). “Non-local neural networks,” in *Proceedings of the IEEE conference on computer vision and pattern recognition*, Salt Lake City, UT, 7794–7803.

[B26] Woo S. Park J. Lee J. Y. Kweon I. S. (2018). “CBAM: Convolutional block attention module,” in *Proceedings of the European conference on computer vision (ECCV)*, Cham: Springer, 3–19. 10.1007/978-3-030-01234-2_1

[B27] Xu P. Xu H. Diao C. Ye Z. (2017). Self-training-based spectral image reconstruction for art paintings with multispectral imaging. *Appl. Opt.* 56 8461–8470. 10.1364/ao.56.008461 29091630

[B28] Yan Y. Zhang L. Li J. Wei W. Zhang Y. (2018). “Accurate spectral super-resolution from a single RGB image using multi-scale CNN,” in *Proceedings of the Chinese conference on pattern recognition and computer vision (PRCV)*, (Cham: Springer), 206–217. 10.1007/978-3-030-03335-4_18

[B29] Zhang J. Sun Y. Chen J. Yang D. Liang R. (2020). Deep-learning-based hyperspectral recovery from a single RGB image. *Opt. Lett.* 45 5676–5679. 10.1364/ol.405061 33057256

